# A clinical presentation of herpangina with ulcerative lesion over uvula

**DOI:** 10.11604/pamj.2024.48.133.43209

**Published:** 2024-07-24

**Authors:** Pawan Banduji Itankar, Gaurav Rajendra Sawarkar

**Affiliations:** 1 Department of Rachana Sharir, Mahatma Gandhi Ayurved College Hospital and Research Centre, Datta Meghe Institute of Higher Education and Research (Deemed to be University) Salod (H), Wardha, India

**Keywords:** Herpangina, ulcerative lesion, fever

## Image in medicine

A 23-year-old male patient came to the outpatient department (OPD) with complaints of an ulcerative lesion over the uvula, fever, headache, and throat pain for 3 to 4 days, and no past medical history. He took antibiotics (azithromycin) and nonsteroidal anti-inflammatory drugs (NSAIDs) (paracetamol+ibuprofen) for three days after taking NSAIDs only the pain subsided but didn´t get relief from the ulcerative lesion. Therefore, an antiviral (Acicylovir) prescribed for five days helps to relieve associated symptoms, and the ulcerative lesion healed completely. Enteroviruses (i.e group A coxsackie virus) are the source of the viral infection known as herpangina, which often strikes children under the age of ten years; though it can strike anybody at any age depending on the immunity of an individual and is seen during autumn and summer season for 7 to 10 days. Clinically, it is an acute self-limiting tiny ulcerative or vesicular lesion in the posterior oropharynx, uvula, and throat region accompanied by an intense fever associated with headache and loss of appetite. Uses of antibiotics are ineffective in such conditions.

**Figure 1:**
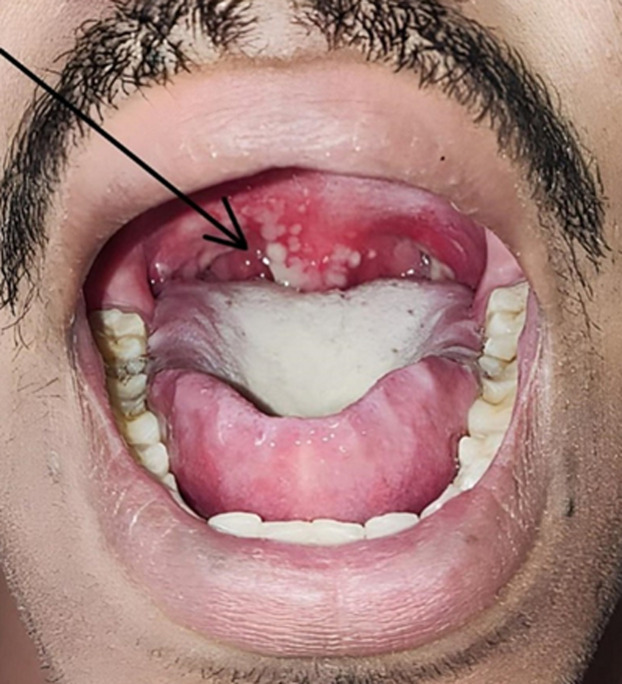
ulcerative or vesicular lesion over uvula

